# A Review of the Role of Paraprobiotics in the Formulation of High-Protein Ice Cream as an Advanced Functional Food

**DOI:** 10.1155/ijfo/8875377

**Published:** 2025-06-17

**Authors:** Seyed Ali Issazadeh, Samaneh Hatami

**Affiliations:** ^1^Department of Food Science and Technology, Quchan Branch, Islamic Azad University, Quchan, Iran; ^2^Department of Food Science and Technology, Faculty of Agriculture, Ferdowsi University of Mashhad, Mashhad, Iran

**Keywords:** anti-inflammatory, extended shelf-life, high-protein ice cream, nonvital bacteria, paraprobiotics

## Abstract

Paraprobiotics are nonvital bacteria that have health benefits. Recently, they have been incorporated into dairy products as supplements to probiotics. The advantages of using paraprobiotics mainly include superior resistance to processing conditions, extended shelf-life, and safer consumption by people with weaker immune systems. In high-protein ice cream, paraprobiotics enhance freezing stability and eliminate the risks of microbial overgrowth and rare adverse effects. They also promote gut health, regulate immune responses, and have anti-inflammatory properties, which are important for children. Moreover, paraprobiotics enhance the functional value of high-protein ice cream by promoting gut health and immune support, making it an ideal functional supplement for athletes and bodybuilders. This review discusses recent developments in high-protein ice cream fortified with paraprobiotics, focusing on product stability, texture, and consumer acceptance while addressing challenges in the sensory quality of the final product.

## 1. Introduction

The concept of functional foods has its roots in long-standing traditions of health promotion through diet, dating back to thousands of years ago and evolving with advancements in science and technology. As the knowledge of food–human body interactions has grown, it has become evident that the nutritional and functional properties of certain foods can be enhanced by incorporating bioactive ingredients. High-protein ice cream fortified with paraprobiotics represents an innovative functional food promoting satiety, supporting muscle growth, and improving metabolism. However, the lactose, fat, and protein contents of traditional ice creams can pose challenges to individuals with lactose intolerance, milk protein allergies, or dietary restrictions [1].

Many dairy products, commonly used as carriers of probiotics, have unfavorable conditions for the survival of paraprobiotics. For instance, the high acidity of yogurt or thermal processes such as pasteurization and sterilization often result in a reduction or complete loss of viable probiotic cells [2, 3]. In the case of ice cream production, the mix is typically subjected to pasteurization rather than sterilization prior to freezing, as sterilization would eliminate all viable microorganisms, including probiotics, which is not desirable for functional ice cream formulations. Given these limitations, the use of paraprobiotics (inactivated probiotics) as alternatives to live probiotics in dairy products such as high-protein ice cream has gained attention. Paraprobiotics offer several advantages, including greater stability during production processes, no need for a cold chain during storage, and no alteration in the sensory characteristics of the product. Furthermore, in contrast to live probiotics, which may pose risks to individuals with compromised immune systems, paraprobiotics provide enhanced safety benefits and reduce the risk of infections associated with the consumption of live probiotics [4].

Numerous clinical studies have been conducted on the use of paraprobiotics in dairy products, which can inform their application in high-protein ice cream. For example, a study on the yogurt containing the paraprobiotic *Lactobacillus gasseri* CP2305 has demonstrated that daily consumption of this product for 5 weeks improves sleep quality and decreases stress symptoms in individuals under psychological pressure [5, 6], suggesting potential benefits that could be explored in paraprobiotic-enriched ice cream.

Additionally, another study indicated that daily consumption of the yogurt containing the inactivated cells of *Lactobacillus delbrueckii* subsp. *bulgaricus*, *Streptococcus thermophilus*, and *Lactobacillus acidophilus* could prevent damage to the intestinal barrier and improve gastrointestinal health [7]. Moreover, research on children revealed that consumption of the fermented milk of cow comprising the inactivated cells of *Lactobacillus paracasei* CBA L74 (as a paraprobiotic) gave rise to the number of the gut useful bacteria like *Faecalibacterium* and *Bacteroides*, which could positively impact gastrointestinal health [8].

Dairy products remain the primary carriers of probiotics and paraprobiotics; however, attention has recently shifted toward incorporating these ingredients into nondairy foods and supplements. This shift is driven by factors such as lactose intolerance, milk allergies, concerns about fat and cholesterol, vegetarian diets, and the presence of antibiotics and hormones in dairy products. Paraprobiotics have the advantage of high stability under various conditions and, unlike live probiotics, can be used in dry foods, beverages, and supplements without losing quality [9, 10].

In a randomized controlled clinical trial by Barros et al. [11], healthy adults consumed 300 mL of a whey–grape juice beverage containing the paraprobiotic *Lacticaseibacillus casei* 01 in single-dose sessions, demonstrating its potential to regulate postprandial glycemia.

Paraprobiotics have also been used in nutritional supplements as alternatives to live probiotics, providing insights applicable to high-protein ice cream formulation. Studies have shown paraprobiotics can effectively enhance the immune system, lower inflammation, improve digestive system function, and alleviate the symptoms of stress and depression. These are the benefits that could enhance the functional properties of paraprobiotic-containing ice cream. For instance, supplementation with heat-inactivated *L. paracasei* MCC1849, daily administered as 100 g of fermented milk for 12 weeks, was found to enhance the body's resistance to viral infections such as the common cold [12]. Additionally, some studies suggest that paraprobiotics may contribute to improved mood states and reduced stress-related symptoms by influencing the gut–brain axis [6].

High-protein ice cream enriched with paraprobiotics is emerging as one of the most significant innovations in the food industry, owing to the growing demand for healthier functional foods. This innovation strikes a balance between the nutritional value and popularity of ice cream as a healthy snack [13].

The development of high-protein ice cream supplemented with paraprobiotics stands out as one of the most important innovations in the food industry, because of the growing demand for healthier functional foods. There is an increased health awareness related to functional foods which could help alleviate a number of health disorders associated with digestion, metabolic disorders, and immune system deficiencies. Paraprobiotics represent a new frontier of functional enrichment of foods. Owing to being viable, biologically active probiotics do not resist any of the issues associated with live microorganisms such as storage problems [14].

Paired with high-protein ice cream, paraprobiotics also enable the manufacturer to market a functional dessert supportive of muscle synthesis and metabolism, along with gut health and immune system function. This is rather important in the wake of increasing lactose intolerance and diets that avoid certain food components, as traditional ice cream contains fat and lactose, both of which decrease its acceptability. The paraprobiotics help in allaying these concerns by offering health benefits similar to those of live probiotics, without the strict storage conditions or any viability concern in variable temperature conditions [6].

More importantly, the development of paraprobiotic-enriched high-protein ice cream fits in with the current paradigm in combating chronic diseases like obesity, diabetes, and cardiovascular diseases, all caused by dietary inputs. This functional ice cream with incorporated paraprobiotics further pushes the health-conscious customer paradigm for potential cholesterol reduction, gut microbiota management, and overall metabolic health [15, 16].

While the formulation and benefits of such products will be analyzed, an effort is made to judge the role of paraprobiotics in converting a conventional convenience food into a health-promoting functional food. Until now, no application of paraprobiotics has been reported in ice cream formulation. This review is aimed at discussing the technologies related to paraprobiotic production, the formulation of high-protein ice cream, and the benefits of incorporating paraprobiotics into such ice cream. It also investigates the impacts of paraprobiotics on gut function and the immune system, ultimately positioning high-protein ice cream as a healthy functional food dessert for athletes.

## 2. Immunological Mechanisms of Paraprobiotics

Paraprobiotics exhibit biological functions similar to live probiotics; nevertheless, since they are inactivated, they do not pose risks such as antibiotic resistance gene transfer and systemic infections. The immunomodulatory and anti-inflammatory properties of paraprobiotics, such as those exhibited by *Lactobacillus helveticus* MIMLh5 and *Lactiplantibacillus plantarum*, primarily depend on the cell surface components like peptidoglycan (PGN), teichoic acids (TAs), and exopolysaccharides (EPSs). These components activate the host immune receptors, including nucleotide-binding oligomerization domain receptors (NODs) and toll-like receptors (TLRs), which regulate cellular signaling pathways to promote immune balance and enhance defense mechanisms [4, 17].

### 2.1. Role of Paraprobiotics in Regulating Innate Immunity and Enhancing Host Defense System

The potential of modulating immune responses and promoting the host health plays a crucial role in assessing the probiotic activity of bacterial strains. At the same time, the precise identification of the components of bacterial cells involved in immune cell interactions remains a challenge [18]. The innate immune system acts as the first defense line of the body against pathogens. Paraprobiotics activate pathogen recognition receptors (PRRs), including TLR2 and NOD2, in immune cells such as dendritic cells and macrophages, stimulating signaling pathways such as mitogen-activated protein kinases (MAPKs) and nuclear factor kappa-light-chain-enhancer of activated B cells (NF-*κ*B). This activation raises the production of proinflammatory cytokines, including interleukin 2 (IL-2), interferon gamma (IFN-*γ*), and tumor necrosis factor-alpha (TNF-*α*), facilitating a rapid immune response to pathogens. Furthermore, paraprobiotics positively influence mucosal and systemic immune function by regulating the expression of immune-related genes [19].

In a project performed by Taverniti et al. [20], *L. helveticus* MIMLh5, originally isolated from Grana Padano cheese whey starter, and its surface-layer protein (SlpA) were examined. The findings indicated that this strain and its SlpA exhibited anti-inflammatory effects by reducing NF-*κ*B activation in intestinal epithelial cancer coli-2 (Caco-2) cells, thereby helping to mitigate inflammation. On the other hand, these compounds stimulated the innate immune system by increasing the expression of inflammatory factors such as TNF-*α* and cyclooxygenase-2 (COX-2) in human macrophages (U937) via toll-like receptor 2 (TLR2). However, SlpA had no effect on the expression of the anti-inflammatory cytokine IL-10. A similar response was observed in the macrophages extracted from the bone marrow and peritoneal cavity of the mice. These findings highlight the crucial role of SlpA in immune stimulation and its potential to enhance the host defense against infections [20]. Furthermore, the results support the idea that immunomodulatory activity is not dependent on the bacterial cell viability, paving the way for the development of safer therapeutic approaches based on paraprobiotics [20].

### 2.2. Role of Paraprobiotics in Immunomodulation and Cytokine Responses

Paraprobiotics such as *L. plantarum* L-137, *L. gasseri* CP2305, and *L. paracasei* CBA L74 exhibit biological mechanisms similar to probiotics, including immunomodulation and gut microbiome modulation; however, due to the incomplete understanding of their mechanisms of action, they are often studied in comparison to live probiotics to elucidate their specific effects [4].

The bacterial cell wall components, namely PGN, TA, and lipopolysaccharide (LPS), significantly influence the biological activity of paraprobiotics. PGN, the major component of the cell wall in Gram-positive bacteria, consists of the polysaccharide chains of N-acetylglucosamine (NAG) and N-acetylmuramic acid (NAM) linked by *β*-1,4 bonds and crosslinked via peptide bridges containing amino acids such as alanine, glutamic acid, and diaminopimelic acid [21]. Variations in the sequence and type of these amino acids among bacterial species result in distinct immunological effects. Similarly, TA are composed of glycerol or ribitol phosphate polymers anchored to the cell wall or membrane, contributing to immune response stimulation [22, 23].

The immunomodulatory activities of paraprobiotics are bidirectional; they can stimulate the production of proinflammatory cytokines including IL-2, IL-12, IL-8, TNF-*α*, and IFN-*γ*, as well as anti-inflammatory cytokines like IL-10, IL-6, IL-4, and IL-13. Additionally, paraprobiotics play an essential role in the production of immunoglobulins, namely, IgE, IgA, and IgG1. Studies have shown that both live probiotics and paraprobiotics induce similar immune responses in the body. Despite strong scientific evidence supporting the biological effects of paraprobiotics, their potential to support general health, such as enhancing immune system function and gut health, requires further research [24].

TA and PGN in the cell wall of certain *Lactobacillus* species, such as *L. plantarum*, are capable of stimulating the production of cytokines like IL-10 and IL-6 which help reduce chronic inflammation and regulate immune responses. On the other hand, when innate immunity requires enhancement, paraprobiotics can promote the generation of proinflammatory cytokines which are effective in combating bacterial and viral infections [25].

The stimulation and modulation of the immune system by the cell wall components such as PGN in probiotics (viable microorganisms) and paraprobiotics (nonviable microbial components or lysates) occur through various mechanisms [4, 17].

In a research project performed by Hatami et al. [26], the impacts of the PGN and cell wall of the probiotics *L. gasseri* 52b (from healthy human vagina), *L. acidophilus* AC2 (from sourdough), *L. plantarum* M11 (from milk), and *Limosilactobacillus fermentum* 19SH (from Horreh, a traditional Iranian fermented food) were investigated on the immune system of mice. The findings revealed that the generation of IL-12 in spleen cells was remarkably more pronounced when exposed to *L. plantarum* M11 cell wall at 110.66 pg/mL and that of *L. gasseri* 52b at 86.53 pg/mL, compared to the other groups. Furthermore, these compounds led to an increase in the production of IFN-*γ* which plays a key role in cellular immune stimulation and tumor growth inhibition. Nonetheless, it was noteworthy that the production of proinflammatory cytokines in *L. plantarum* M11 exceeded normal levels. When this strain was administered live to laboratory mice with (1.5 × 10^8^ CFU/mL (Days 1–14 before tumor injection) and 1.2 × 10^9^ CFU/mL (Days 17–38 after the injection)), instead of improving their conditions, it exacerbated the disease and enlarged the colon tumor. These findings indicated that, in some cases, an excessive immune response can have adverse effects on the treatment process. Therefore, the dosage and type of paraprobiotic bacteria used in dairy and nondairy products require further investigation and research [26].

### 2.3. Paraprobiotics and Gut Barrier Integrity: Immunomodulation and Inflammation Reduction

Retention of the gut barrier integrity plays a crucial role in regulating immune responses and reducing inflammation. The intestinal epithelial barrier prevents the direct contact between microbes and the gut cells via mechanisms like the mucus layer and the production of antibacterial molecules like regenerating islet-derived protein III-gamma (RegIII*γ*), thus restraining excessive immune system activation. Studies have demonstrated that changes in the gut microbiota composition are able to disrupt the function of this barrier and elevate systemic inflammation [27].

In addition, the vagus nerve–dependent inflammatory reflex also has a role in lowering inflammation by inhibiting the production of inflammatory cytokines. This mechanism involves the release of acetylcholine receptors and the activation of alpha7 nicotinic acetylcholine receptor (*α*7nAChR) on immune cells, leading to a decrease in the generation of inflammatory factors including IL-1*β* and TNF-*α*. Furthermore, increased levels of glucocorticoids and melanocyte-stimulating hormone (MSH) in response to neural signals produce further anti-inflammatory effects. These findings suggest that maintaining gut barrier integrity not only prevents the entry of pathogens but also helps reduce inflammation through the regulation of immune–neural pathways [25].

**Figure 1:**
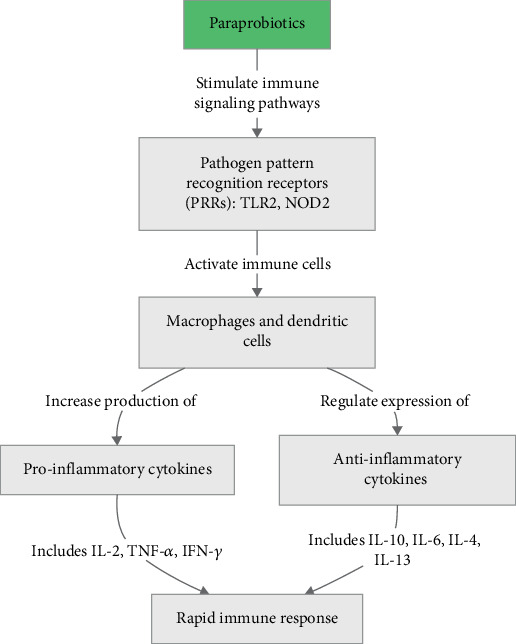
Role of paraprobiotics in modulating the innate immune response through PRRs and cytokine production. The conceptual diagram is designed by the authors based on Section 2 [4, 19–24]. Abbreviations: TLR: toll-like receptor; NOD: nucleotide-binding oligomerization domain-like receptor; IL: interleukin; TNF: tumor necrosis factor-alpha; IFN: interferon-gamma.

Studies have shown that paraprobiotics can diminish intestinal permeability by improving the expression of cell junction proteins such as claudin and occludin [28]. For example, research on *L. gasseri* CP2305 indicated that the daily consumption of this paraprobiotic, administered via a sterilized fermented milk-based beverage at a dose of 10^10^ heat-inactivated bacterial cells per 190 g serving, enhanced the gut barrier function and alleviated the gastrointestinal symptoms associated with stress in healthy medical students [29]. All the steps above are depicted in Figure 1.

## 3. Production Methods of Paraprobiotics

**Figure 2:**
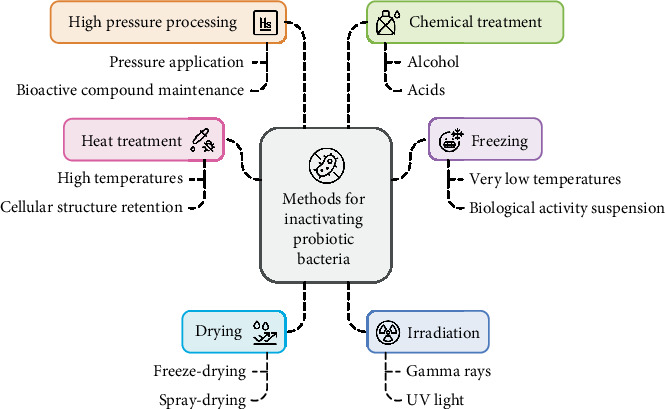
Different inactivation mechanisms of probiotics to produce paraprobiotics.

Isolation has an important role in understanding the health advantages of paraprobiotics as well as their modes of action. For separation, a variety of methods are employed, including techniques of cell disruption, heat treatment, freezing, drying, irradiation with gamma rays or UV light, high-pressure processing (HPP), and chemical treatment [30] (Figure 2). The methods of isolation and further purification should be properly selected to align with the specific characteristics of the studied molecules. The methodologies by which these components are isolated determine their health benefits. It is emphasized that the choice of proper methods and conditions of inactivation will be very important for the effect of paraprobiotics [25].  Table 1 summarizes the studies on inactivation methods and their health benefits.

### 3.1. Selection of Paraprobiotic Production Technique for the Immune System Modulation

As an example of the thermal treatments listed in Table 1, Lee et al. [19] explored the use of a heat inactivation approach for *Bacillus velezensis* GV1. Using this approach, they produced the paraprobiotics of this strain. Paraprobiotics are inactivated microbial cells or cell fractions that confer health benefits such as immune system modulation and gut microbiota regulation. In this study, GV1 refers to heat-killed *B. velezensis* GV1, a paraprobiotic derived from a probiotic strain isolated from fermented foods. The effects of GV1 on gut microbiota and immunology were investigated both in vitro and in vivo. After being treated with the heat-killed *B. velezensis* GV1, RAW 264.7 macrophages (a widely used murine macrophage cell line for in vitro immunological studies) produced more nitric oxide (NO) and secreted higher levels of cytokines including interleukin-6 (IL-6), interleukin-1*β* (IL-1*β*), and TNF-*α* [19].

Furthermore, it reversed the immunosuppressive effects of cyclophosphamide (CTX) by repairing splenic impairment and regaining the immune organ index. While IL-2 was decreased, the immune-related cytokines in the spleen and thymus elevated IL-1*β*, IFN-*γ*, and TNF-*α*. Moreover, the CTX-damaged colon and microbiota were significantly improved by treating with GV1, reducing harmful bacteria like *Stasulfovirichoaceaceae* and *Esulfoviricheaceaceae*, and increasing the relative frequency of advantageous bacterial families like Lactobacillaceae, Akkermansiaceae, and Coriobacteriaceae. In conclusion, the heat-killed paraprobiotic GV1 could be an immunomodulatory compound for application either as a functional ingredient or an immune system-improving drug [19, 31].

The methods of cell membrane protein denaturation seem to represent the best approach for obtaining paraprobiotics. Treatment techniques often directly target the bacterial cell membrane and proteins. These techniques allow the degradation of the bacterial cell wall but preserve the integrity of major immunomodulatory components such as PGNs and fragments from the cell wall. According to Lee et al. [19] and Tsilingiri et al. [37], thermal treatment and ultrasonic disruption are effective due to cell membrane disruption and assurance of no remaining viable cells. Such methods enhance immunity while preserving the bioactive components responsible for the stimulation of the host immune response [19, 37]. Other methods, like UV or gamma irradiation, rely on DNA damage and, therefore, operate in a manner perhaps not effective in the production of paraprobiotics, as such DNA destruction does not necessarily ensure complete bacterial inactivation through the inability to compromise the cell wall structure. While heat killing is a common method of producing paraprobiotics, as demonstrated by Xie et al. [32] for *Lactobacillus rhamnosus* GG (LGG), other methods like UV or gamma irradiation which rely on DNA damage may not ensure the preservation of the cell wall components critical for paraprobiotic function. The authors tested the heat-killed LGG (HK-LGG) on Caco-2 cells in vitro and declared that the intestinal barrier function was restored, which had been impaired by LPS through the myosin light chain kinase/myosin light chain (MLCK/MLC) pathway. This study indicated that both the live and UV-inactivated LGG could reduce IL-8 proinflammatory cytokine in the Caco-2 cells when induced with flagellin at 100 ng/mL, suggesting that inactivation methods may vary in their effects, depending on the intended outcome. Based on these findings, the HK-LGG could potentially serve as a safer alternative in specific cases, particularly given its demonstrated safety at lower concentrations, including 1 *μ*g/mL (10^6^ CFU/mL) [33] and approximately 1.4 × 10^6^ CFU/mL (multiplicity of infection, MOI = 10 : 1) [38], and its protective effects on intestinal epithelial cells (Table 1).

Following the evaluation of inactivation methods for paraprobiotic production and their diverse health benefits such as immune system modulation and gut health enhancement, this section explores their application in high-protein ice cream, focusing on the resulting paraprobiotic properties and technological characteristics essential for functional food development.

### 3.2. Impact of Probiotic Inactivation Methods on Functional and Technological Properties of High-Protein Ice Cream

High-protein ice cream, as an innovative functional food, offers a suitable platform for the incorporation of paraprobiotics derived from probiotic strains such as *L. acidophilus*, *L. casei*, and *B. animalis* [31, 34]. This section examines the impact of inactivation methods (thermal, nonthermal, irradiation, chemical, and supercritical carbon dioxide) on these strains in high-protein ice cream, with an emphasis on preserving bioactive components such as PGN and EPS. Although research is limited on the application of paraprobiotics in high-protein ice cream [6], general data on the inactivation methods [31, 34] and characteristics of high-protein ice cream [39–41] suggest that these methods can enhance the product's functional (e.g., texture, viscosity, and emulsion stability) and technological (e.g., flavor and sensory acceptance) properties.

Thermal treatments, such as pasteurization at 60°C for 40 min or 80°C for 15 min, are suitable to inactivate *B. animalis*, as this strain retains its cell membrane integrity and enzymatic activity [34]. These methods increase viscosity by altering the protein structure of the ice cream matrix, leading to a firmer texture and improved overrun (volume increase) [39, 41]. However, high temperatures (> 80°C) may induce Maillard reactions, resulting in undesirable burnt flavors and reduced sensory acceptance [34]. Thus, mild thermal treatments are recommended so as to preserve sensory quality [39].

Nonthermal methods, including HPP (100–600 MPa) and high-intensity ultrasound (20 kHz, 792 W/cm^2^), minimize thermal damage to bioactive components [34]. HPP, particularly for *B. animalis* and *L. casei*, preserves EPS, which enhances water-holding capacity, resulting in a softer texture and better resistance to ice crystal formation [34, 41]. Ultrasound, especially for *L. casei*, causes minimal damage to the cell membrane integrity and improves the mixture uniformity, creating a creamier mouthfeel [6, 34]. However, excessive acoustic power may induce lipid oxidation, leading to off-flavor [34]. HPP is preferred for premium high-protein ice cream production, owing to its ability to preserve sensory quality and extend shelf-life [39, 41].

Irradiation (gamma rays, 4–6.5 kGy) and chemical treatments (e.g., pH 1 or 12.5) are less suitable for high-protein ice cream, due to the significant degradation of PGN and EPS [34]. Although *L. acidophilus* shows relative resistance, gamma irradiation may produce astringent flavors that reduce sensory acceptance [34]. Extreme pH treatments induce acidic or alkaline flavors which are incompatible with ice cream's sensory profile [34]. These methods are more appropriate for research applications than for industrial production [31].

Supercritical carbon dioxide (8–30 MPa, 40°C) is effective on *B. animalis*, as this strain exhibits high resistance to the destructive effects of the supercritical CO_2_ extraction and preserves bioactive components [34]. This method can improve emulsion stability but requires sophisticated equipment, which may limit industrial scalability [39].

The choice of inactivation method should balance the preservation of bioactive components, sensory quality, and industrial feasibility. Nonthermal methods like HPP and ultrasound are more suitable to produce high-protein ice creams containing paraprobiotics, because of their ability to preserve EPS, enhance texture, and improve sensory quality [34, 39, 41]. Mild thermal treatments also offer a cost-effective option for strains like *B. animalis* [34]. Given the limited research in this area, future studies should focus on optimizing combined methods (e.g., HPP with mild heating) and conducting sensory evaluation to examine consumer acceptance [39–41].

### 3.3. Formulation of High-Protein Ice Cream With Paraprobiotics

The high-protein ice cream discussed in this review is conceptualized as a functional carrier for paraprobiotics, formulated with 5%–7% fat (derived from cream and milk fat), 30%–35% solids-not-fat (SNF), including 15.02%–18.59% protein (sourced from milk protein concentrate (MPC) and whey protein isolate (WPI)), and 35%–40% total solids (TS) comprising sweeteners (e.g., sucralose) and stabilizers (e.g., guar gum). This composition was designed to ensure the stability of paraprobiotics during freezing and enhance nutritional value, aligned with findings on high-protein ice cream formulations [42]. Low-fat ice cream typically contains 0.5%–3% milk fat, with an average of 2.5% [43, 44]. Fat-free ice cream contains less than 0.625% fat [44], and these classifications comply with the Food and Drug Administration (FDA) regulations for reduced-fat, low-fat, and nonfat ice creams (< 10% milk fat). The low-fat variants can be developed by adjusting the fat content to 0.5%–3%, maintaining sensory acceptability [43, 45].

Regarding protein sources, in order to achieve a target of 15% protein, 15% whey protein concentrate (WPC, 80% protein) alone is insufficient, as it contributes approximately 12% protein (15 × 0.8 = 12%) [46]. Therefore, the formulation combines WPC with WPI (≥ 90% protein) and MPC (42%–85% protein), as detailed in Table 2. For example, mixing 10% WPC (~8% protein) with 5% WPI (~4.5% protein) can achieve ~12.5% protein, while increasing the WPI or MPC proportion enables levels up to 15.02%–18.59% (wt.), as defined in Section 5 [52–54]. The use of WPI and MPC aligns with consumer preferences for milk and whey proteins, which are perceived as healthier and more effective in raising the protein content compared to casein or plant-based proteins [39], in addition to supporting the nutritional benefits of high-protein frozen desserts [40].

## 4. Effect of Paraprobiotics on Formulation of Functional Foods

The role of paraprobiotics in the formulation of functional foods like high-protein ice cream is gaining attention, owing to their potential health benefits and technological advantages.

### 4.1. Health Benefits

Paraprobiotics derived from *B. velezensis* GV1 have been shown to improve immune responses and gut microbiota composition, promoting beneficial bacterial families while decreasing harmful ones [19]. They release bioactive compounds such as EPSs and PGN, which have health benefits [55].

Research on the effects of paraprobiotics, particularly their PGN components, has revealed significant benefits for intestinal function and disease treatment. PGN, which is a moiety of the bacterial cell wall, has a substantial effect on immune system stimulation even if the cell is nonviable. In the gut, they help modulate the immune response by PRRs, including TLRs, on the intestinal epithelium. This interaction enhances the generation of anti-inflammatory cytokines, which help maintain gut homeostasis and protect against inflammatory diseases such as inflammatory bowel disease (IBD) and irritable bowel syndrome (IBS) [56]. Additionally, paraprobiotics have shown promise in promoting tight junction integrity, which strengthens the gut barrier and reduces intestinal permeability, a key factor in diseases like leaky gut syndrome. Furthermore, the anti-inflammatory properties of paraprobiotic PGN can assist in lowering the severity of chronic gut inflammation, contributing to the treatment of conditions such as colitis and gastrointestinal infections [57, 58].

TAs account for as much as 50% of the dry weight of lactobacilli cell walls [59]. Owing to its anionic polymer structure, TA can form lipoteichoic acid (LTA) when it is connected to the cytoplasmic membrane by lipid anchors or wall teichoic acid (WTA) when it is covalently bonded to PGN. The immunomodulatory characteristics of TA from various *Lactobacillus* species have been the subject of numerous studies [60].

*L. plantarum* LTA diminished the expression of IL-8 generated by S-[2,3-bis(palmitoyloxy)propyl]-N-palmitoyl-Cys-Ser-(Lys)4 (Pam2CSK4) and had an anti-inflammatory influence on the epithelial cells of the human intestine. In addition, this LTA exhibited anti-inflammatory activity in those of the pig intestine. The anti-inflammatory impacts of LTAs vary depending on the species or strain. For instance, Noh et al. [61] indicated that most of the immunomodulatory properties of *L. plantarum* TA relied on D-alanylation [61].

In addition to PGN, other cell wall components like polysaccharides also have a considerable effect on the functionality of paraprobiotics. Polysaccharides are commonly found on the surface of Gram-positive bacteria such as *Lactobacilli*. EPSs are the most well investigated polysaccharides, which may improve bacterial interactions with the surrounding medium, moderate adhesion qualities, preserve against infections, and function as a protective layer [62]. Nikolic et al. [63] claimed EPS produced from numerous *Lactobacillus* species had the capability of altering mucosal and systemic immune responses, bringing about health promotion [63]. EPS purified from *L. rhamnosus* RW-9595M suppressed macrophages by raising IL-10 levels while decreasing IL-6, TNF-*α*, and IL-12 levels. Furthermore, *L. plantarum* BGCG11, generating EPS, demonstrated anti-inflammatory effects, alluding to the immune-suppressive effect of EPS [64]. The acidic moiety of the EPS generated by *L. plantarum* reduced the generation of proinflammatory cytokines (IL-8, IL-6, and MCP-1) in the epithelial cells of the pig intestine in response to exposure to enterotoxigenic *Escherichia coli* (*E. coli*) (ETEC). Aside from its anti-inflammatory properties, EPS is also able to give rise to the immunological response. The EPS purified from the yogurt fermented with *L. delbrueckii* subsp. *bulgaricus* OLL1073R-1 stimulated the generation of IFN-*γ* and activated the mice's natural killer (NK) cells, contributing to the antiviral activity. In addition, EPS is capable of controlling energy metabolism in the host body. The EPS extracted from LGG lowered adipogenesis and the levels of cholesterol ester and triacylglycerol in the serum and liver of mice [65].

### 4.2. Technological Advances

Paraprobiotics have several key technological advantages over live probiotics, particularly in the formulation of high-protein ice cream, as they can be added to the ice cream without concerns for survival during processing, storage, and distribution. While live probiotics are susceptible to heat, high pressure, and acidic conditions, paraprobiotics remain stable and retain their functional properties under adverse conditions including high temperatures, HPP, low pH, and long storage times [14, 66]. Specifically, this resilience provides a wide range of applications varying from conventional food and beverages to functional foods, because it does not require refrigeration and careful handling typical of live cultures.

Furthermore, paraprobiotics enhance the functional value of food products by providing health benefits such as immune system modulation and gut health improvement, similar to live probiotics, but with greater stability. If viable probiotics are taken in wrong proportions, they will cause problems such as bacterial overgrowth or loss of viability during industrial processes [14], whereas nonviability and versatility turn paraprobiotics into an excellent opportunity for producers to develop functional foods with health benefits such as immune system modulation and gut health improvement, which can be easily incorporated into the food basket of consumers. It is important to note that paraprobiotics enhance the functional properties of food products through bioactive compounds, including PGN and EPS, rather than giving rise to their nutrient content (e.g., proteins and vitamins) [6, 14]. Beyond enhancing gut health and immune system function, paraprobiotics can be combined with high-protein formulations, including innovative food products like ice cream, to further elevate their functional health benefits and meet diverse dietary requirements.

## 5. Importance of High-Protein Ice Cream

Protein is a crucial macronutrient needed for tissue structures and the control of many physiological processes. In addition to providing approximately 4 kcal/g, protein is necessary for body growth. For men weighing about 65 kg, the recommended daily intake of protein is 54 g, and adjusting the protein intake based on individual needs is essential for optimal performance and recovery. Athletes typically require 1.2–2.0 g of protein per kilogram of body weight per day, depending on their training and goals [67].

High-protein ice creams with reduced sugar or sweetened with natural sweeteners serve as a dietary supplement for athletes and a potential dessert option for individuals with conditions like diabetes, provided the formulations meet low-glycemic requirements. The growing consumer interest in high-protein frozen desserts is evidenced by commercial products such as Breyer's Delights, Chilly Cow, Enlightened, Halo Top, and Skinny Cow, which are perceived to be healthier due to their elevated protein content, with milk protein, whey protein, and ultrafiltered milk being preferred over casein or plant-based proteins [39]. These products cater to the increasing demand for functional foods that combine nutritional benefits with enjoyable sensory attributes [40].

High-protein frozen desserts are recognized for meeting the nutritional needs of specific populations such as those requiring increased protein intake [40]. Beyond dairy-based proteins like MPC [68, 69] and WPI [70], alternative sources such as colostrum powder [71], pea protein isolate (PPI) [72], and spirulina in frozen yogurt [73] have been explored, typically achieving moderate protein levels (4.6%–7.18%), compared to advanced formulations reaching approximately 15.02%–18.59% (wt.) [45].

In this review, high-protein ice cream is defined as a type of ice cream containing approximately 15.05%–18.59% (wt.) protein based on experimental formulations utilizing WPI, which is significantly higher than that of traditional ice cream (typically 2.6%–4.6% wt.). This classification is supported by industry trends rather than specific regulatory standards, as no minimum protein content is mandated for this category by regulatory bodies like the FDA or EFSA, even though nutritional claims are governed by regulations such as EU No. 1924/2006 [45, 53]. High-protein formulations, which are often combined with paraprobiotics, are appropriate for muscle building due to the high biological value of whey proteins and support immune system health through paraprobiotic components [65].

High-protein ice cream has gained popularity in recent years, with various protein sources employed to enhance its nutritional profile. The selection of protein ingredients significantly influences both the nutritional value and functional properties of the final product. Table 2 presents the compositional profiles of key high-protein dairy powders commonly used in ice cream formulation, including WPC, WPI, micellar casein concentrate (MCC), MPC, and colostrum powder, based on the data compiled from the literature [47, 48].

The literature highlights several key protein sources utilized in the formulation of high-protein ice cream. For example: (a) WPI: studies show that incorporating WPI significantly increases the ice cream protein content (up to 10%). This high-protein additive enhances texture and consistency, owing to its functional properties, but may negatively affect sensory attributes such as melt score due to elevating the matrix density [50].

(b) MPC: this ingredient has been shown to closely simulate the sensory properties of traditional ice cream while raising the protein content by approximately 5%–7% (*w*/*w*). It also contributes to better structural stability and lower melting rates, compared to other protein sources such as soy and pea proteins. Before discussing other protein sources, it is worth noting that milk protein consists of casein and whey proteins, which possess numerous nutritional and functional properties. Advancements in technology have enabled the separation and purification of specific types of dairy proteins to high levels, meeting the technological requirements of food systems. Caseinates, coprecipitates, rennet casein, and acid casein are conventional high-protein dairy powders. Consequently, WPI and WPC have become highly popular because of their versatility and functionality [74, 75]

(c) MCC powder and MPC are recent additives. Membrane and ion-exchange technologies have enabled the manufacture of such specific high-protein compounds, most of which are employed as specialized additives and offer food functionality including solubility, viscosity, gelation, emulsification, and foaming [76]. Drinks, meat systems, bakery products, fermented dairy products (cheese and yogurt), ice creams, coffee creamers, nondairy whiteners, and many other products are prepared with these protein powders. Building on these technological advancements in dairy protein production, the application of protein powders such as MPC, WPI, and MCC is particularly significant in formulating high-protein ice cream, enhancing its nutritional and functional properties. These protein powders are ideal choices due to their ability to increase the protein content (up to 15.02–18.59 wt.% based on experimental and industry standards) [45], improve texture, enhance melting stability, and elevate nutritional value. For instance, WPI, with its high protein purity and good solubility, contributes to improved consistency and muscle recovery, while MPC provides structural stability and a lower melting rate [49, 50]. The primary challenge in using these powders lies in maintaining a desirable flavor and preventing off-flavor in the ice cream, which requires careful formulation optimization. In addition to dairy-based proteins, plant-based protein sources such as PPI and soy protein isolate (SPI) have also been explored for high-protein ice cream formulation to address dietary restrictions and allergies [74, 75, 77]

(d) Plant-based proteins: owing to their enhanced oil and water binding capacities, PPI and SPI have been investigated as alternatives to dairy-based proteins, specifically WPI and MPC, in high-protein ice cream formulations. However, compared with these dairy proteins, PPI and SPI are less flavorful and creamy [74]

Cow's milk protein allergy (CMPA), affecting 2%–6% of infants and 0.16%–0.49% of adults, may cause gastrointestinal distress or anaphylaxis, necessitating dairy-free formulations [78]. This drives demand for plant-based proteins like soy and pea. When processed via fermentation or extrusion, such proteins offer amino acid profiles comparable to those of dairy proteins, with a protein digestibility-corrected amino acid score (PDCAAS) of ~0.9 [48, 49]. Noting plant protein allergies, such as soy (~0.1%–0.4% prevalence), clear allergen labeling is essential [78–80]. Paraprobiotics (Section 6) enhance safety and appeal for allergy-affected consumers, expanding the high-protein ice cream market. Despite these alternatives, dairy proteins like whey remain prevalent for their sensory and nutritional benefits.

(e): WPC: it has been noted for its nutritional benefits and has been effectively applied to increase protein content while maintaining desirable physicochemical properties [81, 82].

Recent studies have also focused on functional ice creams enriched with whey protein gels and blueberries which, respectively, give rise to protein content and bioactive compounds while improving health benefits [83].

In summary, while various protein combinations have been used to produce high-protein ice cream, the choice of the protein source markedly affects the sensory and physical properties of the product. However, a challenge remains in balancing the protein content with flavor and texture, especially when using plant proteins.

Sivasankari et al. [84] examined the physical and sensory properties of ice cream enriched with pulse protein concentrate (PPC) derived from red gram and Bengal gram. Their results showed that the ice cream had a markedly higher protein content (11.76/100 g), compared with the control ice cream (4.90/100 g). It demonstrated that PPC could raise the ice cream nutritional value. Moreover, the sensory evaluation, which was performed on a 9-point hedonic scale, revealed that the ice cream with 5% PPC received a higher overall acceptability score (8.7 for red gram and 8.8 for Bengal gram) than that with 10% PPC (8.4 for red gram and 8.5 for Bengal gram). The physical properties of the ice cream were also evaluated. Incorporation of PPC into the ice cream resulted in a darker color compared to the control one, which was lighter. The pH values of the ice cream samples with 10% PPC showed a slight increase compared to the 5% level. However, there was no significant difference in the acidity values of the experimental and control samples. The findings of this research revealed that PPC may be effectively incorporated into ice cream formulation and provide a nutritious product with desirable sensory properties [84].

Functional dietary components help to naturally prevent illnesses including digestive disorders, obesity, high cholesterol, and diabetes. As a result, bioactive substances such as paraprobiotics are frequently employed to provide food with functional characteristics. Dairy products including ice cream and yogurt are among the best foods for imparting useful characteristics. The fat content of these products may readily be lowered and enhanced with bioactive substances [85, 86].

Table 3 provides data on high-protein diet-friendly ice creams that are aimed at combining nutritional value with health benefits.

## 6. Impact of Paraprobiotics on High-Protein Ice Cream Production

The proteins used such as WPI, casein, and plant proteins like pea and soy, among others, are those that have been known to present benefits in muscle recovery, satiety, and nutrition enhancement in general. As proved by Salles et al. [90], whey and casein proteins are generally added to functional foods to bring about muscle synthesis and satiety, while soy and pea proteins contain a cholesterol-lowering effect and present options for vegan and lactose-intolerant consumers [90].

Conventional ice creams comprise low protein levels (2–5 wt.%), mainly derived from milk solids, that are insufficient to meet elevated nutritional demands. To address this, high-protein ice creams have been developed using protein powders such as WPI and MPC, achieving protein contents of 10%–18.59% (wt.). Although 10% (wt.) protein is commonly achievable in commercial formulations, experimental formulations in this review (Table 3) demonstrate protein levels of 15.02%–18.59% (wt.), as defined based on empirical and industry benchmarks. Adding such high levels of protein to these ice creams supports muscle recovery and weight management. Research confirms such formulations meet dietary protein requirements, particularly for postworkout recovery [53]. Therefore, the fat content is moderated, balanced with the protein content, to help maintain satiety and ensure the product remains enjoyable [90, 91]. According to the literature, such maintenance of a balance allows high-protein ice creams to be considered both functional food and a healthy snack that appeals to a fitness-conscious consumer while controlling total caloric intake [92].

Inclusion of paraprobiotics in functional high-protein foods such as high-protein ice creams can be linked to their potential benefits in managing chronic diseases, for example, improving gut health and management of IBD. Dietary proteins and sugars in ice cream are formulated in a way that will ensure health in the digestive system or modulate the immune system to solve problems such as intestinal inflammation and metabolic syndrome [93]. Studies have shown that paraprobiotics such as fragmented *Lactobacillus amylovorus* CP1563 can reduce body fat, improve lipid and glucose metabolism, and contribute to the prevention of metabolic syndrome in overweight individuals, potentially lowering the risks of chronic diseases including obesity, Type 2 diabetes, and cardiovascular diseases [94].

### 6.1. Effect of Paraprobiotics on the Sensory and Structural Characteristics of Ice Cream

Research has revealed the addition of bioactive compounds derived from probiotics to high-protein ice cream can affect textural properties (e.g., firmness, viscosity, and emulsion stability) and sensory attributes (e.g., taste, aroma, and mouthfeel). Due to their lack of need for viability in the final product, paraprobiotics can be a suitable option for products like ice cream, which are stored under freezing conditions where maintaining the survival of live microorganisms is challenging.

Studies on probiotic ice creams have indicated that the inclusion of probiotics can result in a softer and more uniform texture while improving sensory acceptance. This effect is principally due to the production of EPS by probiotics, which act as emulsifying and stabilizing agents, thus reducing ice crystal sizes and enhancing creaminess [95]. Paraprobiotics, like the inactivated cells of *L. casei* and *Bifidobacterium adolescentis*, are likely to exhibit similar effects, as their structural components, including EPS, can serve as texturizing and stabilizing agents [95, 96]. Additionally, microencapsulation with high-amylose starches (e.g., Hylon) and coatings like chitosan or poly-L-lysine creates small spherical microcapsules (< 100 *μ*m) that prevent a gritty texture and contribute to a smoother mouthfeel [95].

The conceptualized formulation, with a TS content of 35%–40%, a high SNF content (30%–35%), and 10%–12% protein, was designed to enhance the melting resistance and structural integrity of high-protein ice cream. This composition, particularly the elevated protein content (10%–12%), contributed to the improved viscosity and stability of paraprobiotics within the frozen matrix, aligning with observations by [45, 52] on the role of proteins in enhancing ice cream quality. Although this protein level is below the 15.02%–18.59% (wt.) threshold defined in this study (Section 5), it balances nutritional benefits with sensory acceptability, ensuring a softer texture and uniform consistency as reported in studies concerning probiotic ice cream [97]. These attributes underscore the suitability of the formulation as a functional carrier of paraprobiotics while boosting the nutritional value for health-conscious consumers.

### 6.2. Advantages of Paraprobiotics and Disadvantages of Probiotics in Ice Cream

Paraprobiotics have attracted noteworthy attention in the dairy industry for their potential applications as alternatives for traditional probiotics. Rather than presenting the idea of paraprobiotics, it will be put forward as well as the use of probiotics to gain added benefits [98].

Application of paraprobiotics in ice cream provides several advantages associated with stability, safety, and health promotion. In contrast to live probiotics, paraprobiotics are nonviable inactivated probiotic cells that can still produce health-promoting effects such as gut health improvement and immune system modulation. These features make them highly appropriate for ice cream where maintaining the viability of live probiotic bacteria is challenging under freezing conditions and during long-term storage. Their stability ensures that the added value of such preparations is retained in extreme conditions, making them ideal for frozen desserts. Although specific data on paraprobiotic cell density is limited, studies on probiotics suggest a minimum therapeutic threshold of 10^6^–10^7^ CFU/g for health benefits [99]. A similar cell density range is proposed for paraprobiotics to ensure their efficacy in ice cream, pending further research.

As explained by Szydłowska et al. [100], the use of paraprobiotics will exclude the risk of overgrowth or contamination that is sometimes associated with the use of live probiotics [100]. This enhances safety, particularly for severely immunocompromised consumers, by eliminating the rare risk of bacterial translocation and infection reported in exceptional cases with live probiotics. Furthermore, paraprobiotics remain stable under environmental stresses such as temperature and oxygen [101–103].

In addition, paraprobiotics are not affected by storage conditions such as pH and temperature that normally influence the viability of living probiotic cells. This will ensure that the quality and health benefits of the products are maintained over time. The incorporation of paraprobiotics into ice cream gives the manufacturer an avenue to participate in the developing market of functional foods for health-conscious consumers seeking added health benefits to the basic nutrition [14]. Thus, applying paraprobiotics contributes not only to health benefits but also to product stability. For this reason, they are suitable for use in the formulation of ice creams. The Brazilian ice cream market is projected to grow by 37% from $71.52 billion in 2021 to $97.85 billion in 2027, driven by the increasing demand for functional foods [104]. New developments in the ice cream industry are significantly contributing to this growth by addressing consumers' demand for functional and sustainable products. High-protein ice creams fortified with paraprobiotics, as discussed in this study (Sections 3.2 and 6), enhance gut health and immune system function, appealing to athletes and health-conscious consumers while facilitating storage and distribution owing to their stability under freezing conditions [15, 19]. Despite the nutritional benefits of high-protein ice cream, excessive consumption may pose challenges. High protein intake, in particular from concentrated sources like whey or casein, can lead to digestive issues such as bloating, flatulence, or discomfort in some individuals, especially those with lactose intolerance or sensitive digestive systems [105]. However, the satiety induced by the high protein content typically limits overconsumption in most consumers, decreasing the likelihood of such adverse effects [106].

Excessive intake may still occur in specific consumers, including athletes or individuals following high-protein diets, who may consume large quantities to meet their own nutritional goals, potentially increasing the risk of digestive discomfort [107]. Paraprobiotics, which do not contribute to the protein content, can mitigate some digestive concerns by promoting the gut health without requiring excessive intake [100]. Additionally, in July 2019, Perfect Day introduced sustainable ice creams using lab-grown dairy proteins produced via microbial fermentation, which sold out in the United States in less than a day, reflecting the strong consumer interest in eco-friendly innovations. Partnering with Dairy Management Inc., Perfect Day's innovative approach demonstrates scalability, poised to revolutionize sustainable ice cream production [107]. These innovations, complemented by marketing strategies like transparent labeling, expand the market by attracting diverse consumer groups, fueling significant growth [104].

The incorporation of live probiotics into ice cream has garnered attention for their potential health benefits; however, several studies highlight significant challenges related to the product quality and consumer acceptance, which paraprobiotic-containing ice creams are aimed at addressing. A primary issue is sensory modifications caused by the metabolites produced by probiotics. Probiotics generate organic acids such as lactic and acetic acids, particularly during fermentation, which can impart sour or vinegary flavors, lowering sensory acceptance. For instance, *Bifidobacterium* species produce acetic acid, resulting in a vinegary flavor that receives lower sensory scores [108]. Similarly, ice creams made with *Lactobacillus reuteri* exhibit a sourer taste, compared to those with *L. acidophilus*, *L. rhamnosus*, or *Bifidobacterium bifidum*, due to more lactic acid production [109]. Moreover, volatile compounds like acetaldehyde and diacetyl, as well as bitter peptides from proteolytic activity, can introduce off-flavor or unexpected aromas misaligned with consumer expectations of traditional ice cream [97, 110].

Turkmen et al. [111] demonstrated that ice creams with higher kefir content, and thus increased acidity, received lower flavor scores, consistent with the findings linking elevated acidity to decreased sensory quality [111]. Probiotic stability also poses challenges. Although probiotics can sometimes survive in frozen environments, their viable counts often decrease over time, particularly under acidic conditions, impacting their health-promoting efficacy [112]. On the other hand, this reduction in probiotic count does not typically influence the texture of ice cream, as texture is mainly determined by the ice cream matrix including proteins, fats, emulsifiers, and stabilizers, rather than the microbial load [113].

Although certain probiotic metabolites, such as EPS produced by strains like *L. rhamnosus*, may enhance viscosity or creaminess in some formulations [104], some researchers, for example, Mohammadi et al. [114], have realized that supplementation of ice cream with probiotic bacteria has little effect on its flavor, texture, or other sensory characteristics, indicating that careful strain selection can minimize the sensory alterations caused by probiotic fermentation. The loss of viability itself does not directly alter textural properties. Health concerns, such as gastrointestinal risks from excessive probiotic consumption, may deter consumers with specific sensitivities. These challenges necessitate careful strain selection, formulation optimization, and targeted marketing to balance health benefits with consumer expectations [106, 115].

**Figure 3:**
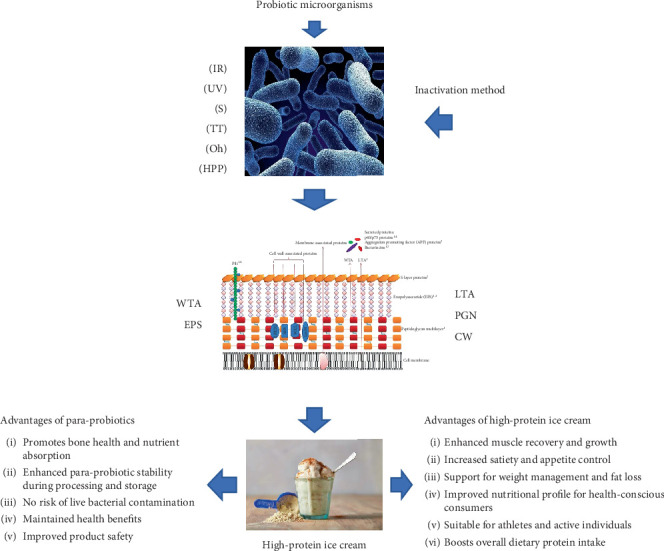
Benefits of paraprobiotic high-protein ice cream. This figure illustrates the advantages of incorporating paraprobiotics and high-protein ingredients into ice cream. Paraprobiotics, produced using inactivation methods (e.g., thermal treatment (TT), ohmic heating (Oh), irradiation (IR), high-pressure processing (HPP), sonication (S), and ultraviolet (UV)), ensure stability during processing and storage while preserving bioactive components like PGN, lipoteichoic acid (LTA), wall teichoic acid (WTA), EPS, and cell wall (CW). These components promote nutrient absorption (e.g., calcium for bone health), immune system modulation, and product safety by eliminating the risks of live bacterial contamination. High-protein ingredients, such as WPI and MPC, enhance the ice cream nutritional profile, supporting muscle synthesis, satiety, and weight management. The content is original, designed by the authors.

In contrast, paraprobiotics, being inactivated, do not ferment and thus avoid producing undesirable metabolites like organic acids or volatile compounds. This preserves the traditional ice cream sensory attributes, including sweetness and uniform texture. Moreover, paraprobiotics eliminate the risks of microbial overgrowth or rare complications associated with live probiotics, making them safer for consumers with compromised immunity [100, 114]. Their structural components, such as polysaccharides, act as texture stabilizers, enhancing the ice cream softness and uniformity [104]. These advantages position paraprobiotics as an ideal choice to formulate high-protein ice cream that offers both high nutritional value and alignment with the growing demand for health-oriented functional foods (Table 4 and Figure 3).

## 7. Conclusions

Incorporating paraprobiotics into high-protein ice cream formulations represents a revolutionary approach to producing functional foods that combine sensory pleasure with health benefits such as improved gut health and immune system modulation. Unlike live probiotics, paraprobiotics exhibit superior stability during freezing and long-term storage, preserving functional properties like texture, viscosity, and emulsion stability, making them ideal for frozen desserts where the retention of live probiotics' viability is technologically challenging. Inactivation methods, such as mild thermal treatment (pasteurization at 60°C–80°C), HPP (100–600 MPa), and high-intensity ultrasound (20 kHz), play a critical role in preserving bioactive components like EPS. These methods enhance the ice cream texture, with thermal treatment increasing viscosity and firmness, HPP providing a softer texture and better resistance to ice crystal formation, and ultrasound creating a creamier mouthfeel and improved uniformity, all of which elevate sensory acceptance. The inactivated nature of paraprobiotics eliminates the risks of microbial overgrowth or rare adverse effects, enhancing safety for consumers with compromised immunity or at health risk. Furthermore, the addition of high protein content significantly enhances the nutritional value of ice cream, aligning with the growing consumer demand for healthier dessert options tailored to diverse dietary needs. WPI which is valued for its high protein purity, excellent solubility, texture enhancement, and nutritional benefits like muscle recovery and satiety and MPC which provides both casein and whey proteins for structural stability and enhanced nutritional value are optimal choices for these formulations. This review underscores the potential of high-protein ice cream containing paraprobiotics as an innovative functional food, highlighting the opportunities for further research to optimize the formulations, enhance sensory properties, and meet market demands.

## Figures and Tables

**Table 1:** Different inactivation methods of probiotics and effects of paraprobiotics on health.

**Inactivation method category**	**Specific method**	**Conditions**	**Probiotic name**	**Function (health benefits obtained)**	**Reference**
Thermal methods	Thermal heating	60°C for 40 min, 80°C for 15 min, or 100°C for 8 min (after the suspension core reaches the target temperature, it is cooled in an ice bath)	*L. acidophilus*, *L. casei*, *Bifidobacterium animalis*	Immunomodulation and anti-inflammatory	[31–34]
	Ohmic heating	Voltage gradient 12.5–15 V/cm, frequency 50–60 Hz, final temperature 80°C–85°C for 5.82–6.12 min (for 12.5 V/cm) or 3.73–3.93 min (for 15 V/cm), immediate cooling to 4°C in an ice bath	*L. casei* 01	Blood sugar control	[4, 11, 34, 35]

Mechanical methods	Sonication	Frequency 20 kHz, acoustic power 792 W/cm^2^, duration 40–120 min (40 min for *L. casei*, 60 min for *L. acidophilus*, 120 min for *B. animalis*)	*L. acidophilus*, *L. casei*, *B. animalis*	Cholesterol reduction	[31–34]
	High pressure	100–600 MPa (200–400 MPa for vegetative bacteria inactivation), temperature 5°C–65°C, holding time 0.01–4 min (depending on the microorganism and the food matrix); for PATP/PATS: 600–800 MPa, 90°C–120°C	Various *Lactobacillus* species	Immunomodulation and anti-inflammatory	[31, 32, 34, 36]

Radiation-based methods	Gamma irradiation	4–6.5 kGy (4 kGy for *L. casei*, 5.5 kGy for *B. animalis*, and 6.5 kGy for *L. acidophilus*) in ice using a cobalt-60 irradiator	*L. acidophilus*, *L. casei*, *B. animalis*	Gut barrier protection	[31, 32, 34]
	pH changes	pH 1 for 30–100 min, pH 12.5 for 50–120 min, or pH 12.75 for 50 min (pH 1/30 min for *B. animalis*, pH 12.75/50 min for *L. casei*, and pH 12.5/120 min for *L. acidophilus*)	*L. acidophilus*, *L. casei*, *B. animalis*	Immunomodulation, antitumor	[34]

Chemical methods	Solvent extraction	Ethanol or acetone 50%–70% for 1–2 h	Various *Lactobacillus* species	Antimicrobial	[31, 32, 34]
	Enzymatic extraction	Lysozyme or protease (e.g., pepsin or trypsin), pH 6–7, 37°C, 1–4 h	Various *Lactobacillus* species	Immunomodulation, antitumor	[31, 32, 34]

Other methods	Supercritical CO_2_ extraction	8–30 MPa, 40°C, 60–180 min (60 min for *L. acidophilus* and *L. casei* at 30 MPa, 180 min for *B. animalis* at 30 MPa), pressure reduction rate 0.5–1 MPa/min	*L. acidophilus*, *L. casei*, *B. animalis*	Immunomodulation and antimicrobial	[4, 11, 34]

*Note:* The following units and abbreviations are used in the conditions' column: min: minute, a unit of time. °C: degrees Celsius. V/cm: volts per centimeter, a unit of electric field strength used in ohmic heating. Hz: hertz, a unit of frequency. kHz: kilohertz, a unit of frequency (1 kHz = 1000 Hz). W/cm^2^: watts per square centimeter, a unit of power density used in ultrasound. MPa: megapascal, a unit of pressure (1 MPa = 10^6^ Pascals). PATP/PATS: pressure-assisted thermal processing/pressure-assisted thermal sterilization, high-pressure techniques combined with elevated temperatures. kGy: kilogray, a unit of absorbed radiation (1 kGy = 1000 Gray) used in gamma irradiation. pH: potential of hydrogen, a measure of acidity or alkalinity, ranging from 0 (acidic) to 14 (alkaline).

**Table 2:** Compositional profiles of high-protein dairy powders used in ice cream formulation.

**Protein powder**	**Protein content (% ** **w**/**w)**	**Fat content (% w/w)**	**Lactose/carbohydrate content (% w/w)**	**Key characteristics**	**Source**
WPC	80%	1%–7%	4%–8%	Enhances texture, improves sensory attributes, good solubility	[47–49]
WPI	≥ 90% (tested as 90.8%)	1.0%	0.6%	Higher protein purity, lower fat and lactose content, excellent for muscle recovery	[48, 50]
MCC	80%–85%	1%–2%	2%–5%	Slow digestion, provides sustained amino acid release, improves creaminess	[47]
MPC	42%–85% (tested as 82.9%)	1.4%–1.5%	3.3%–46% (tested as 3.3%)	Contains both casein and whey, enhances nutritional profile and texture	[49, 50]
Colostrum powder	50%–70%	2%–5%	15%–20%	Rich in immunoglobulins, supports immune system function, lower protein content than the other protein sources	[51]

Abbreviations: MCC, micellar casein concentrate; MPC, milk protein concentrate; *w*/*w*, weight by weight; WPC, whey protein concentrate; WPI, whey protein isolate.

**Table 3:** Formulations of high-protein ice cream.

**Protein**	**Formulation**	**Salient findings**
WPC: 1%–7%	Sugar: Corn syrup and sucrose Fat: 0.1%–7.0% Lactose: Less than 4% Whey protein:casein ratio: 1:0.5–1:4.0 Additional ingredients: Desserts also contain 20%–25% nonfat milk solids, which contribute to the overall texture and flavor	✓ Mimics full-fat ice cream organoleptic attributes ✓ Keeps fat and calories low ✓ Offers a healthier option [87, 88]

WPC: 8%–15%	Sugar: Different types of carbohydrates (6%–14%) Fat: 1%–5%	✓ The dessert is designed to have a protein content ranging from 8% to 15% ✓ Supports muscle maintenance ✓ Enhances satiety ✓ Ideal for high-protein low-calorie intake [87, 88]

Milk protein: 8% (WPC)	Sugar: Beet sugar (13%) and dextrose-equivalent corn syrup solids (4% DE 42) Fat: Milk fat (9.56%)	✓ Ash and lactose decrease as protein rises ✓ Increases viscosity and requires specific processing (e.g., high-RPM blending) to ensure uniformity, resulting in low overrun for a denser texture ✓ Impacts nutritional profile ✓ 8% protein preferred for satiety [87, 88]

First formulation: Protein: 0%–11% (*w*/*w*) (WPC and/or WPI)	Sugar: 10%–20% (*w*/*w*) Fat: 0%–20% (*w*/*w*)	✓ Blends traditional ingredients with protein ✓ Appeals to a wider health-focused market ✓ Probiotic yogurt enhances taste appeal [41, 87]
Second formulation: Protein: 5%–15% (*w*/*w*) (WPC and/or WPI)	Sugar: 10%–20% (*w*/*w*) Fat: 0%–20% (*w*/*w*) Additional ingredients: The formulations also include other components such as flavoring agents (0%–10% *w*/*w*), milk solids (10%–15% *w*/*w*), yogurt (5%–20% *w*/*w*), and water (50%–65% *w*/*w*)	

Whey protein: 4, 6, 8, and 10% (*w*/*w*) (WPC and/or WPI)	Skimmed milk powder (SMP) as the main source of milk protein, which also contains lactose, a sugar	✓ Higher SMP percentage boosts protein content ✓ Causes low overrun and increased hardness ✓ Requires careful ingredient balance [41, 87]

Whey protein: 8%–15% (WPC and/or WPI)	Sugar: 15% (*w*/*w*) Fat: 0%–20% Protein: 3% (*w*/*w*) Flavorings: 0%–10% (*w*/*w*) Milk solids: 10%–15% (*w*/*w*) Yogurt: 0%–25% (*w*/*w*) Water: 50%–65% (*w*/*w*)	✓ Optimized process increases product quality and texture stability ✓ Ensures quality and stability ✓ Adaptable to various frozen desserts ✓ Meets diverse consumer preferences [87, 88]

Derived from milk, especially ALAPLEX protein (MPC, ALAPLEX 1235, 92.6% (*w*/*w*) protein): Formula 1: 9% protein Formula 2: 10% protein Formula 3: 11.5% protein Formula 4: 10% protein	Sugar: 15% (*w*/*w*) Fat: 0%–20% Protein: 3% (*w*/*w*, base protein content before ALAPLEX addition) Flavorings: 0%–10% (*w*/*w*) Milk solids: 10%–15% (*w*/*w*) Yogurt: 0%–25% (*w*/*w*) Water: 50%–65% (*w*/*w*)	✓ Ingredients are mixed in specific ratios ✓ Achieves desired texture and taste ✓ Maintains low-calorie profile [89]

*Note:* High-protein ice cream is defined in this study as a type of ice cream containing 15.02%–18.59% (wt.) protein, based on experimental and industry benchmarks; the formulations listed reflect the variations explored in the literature and practice. ALAPLEX (ALAPLEX 1235) is a MPC brand with a protein content of 92.6% (*w*/*w*), comprising both casein and whey proteins at a ratio similar to milk (approximately 80:20 casein:whey), as reported in [89]. No specific WPI or WPC was used separately in these formulations. The total protein content (9%–11.5%) in the final ice cream product was achieved by incorporating ALAPLEX as the primary protein source. The 3% (*w*/*w*) protein in the formulation column refers to the base protein content of the product before the addition of ALAPLEX. The dextrose equivalent (DE) for corn syrup solids (CSSs) is assumed to be 42, according to common industry standards for frozen dessert formulations, as specific DE values were not provided in the referenced patent [87]. ALAPLEX protein (a comprehensive, milk-derived protein complex).

Abbreviations: RPM, revolutions per minute; SMP, skimmed milk powder; *w*/*w*, weight by weight; WPC, whey protein concentrate.

**Table 4:** Comparison of paraprobiotics and live probiotics in ice cream.

**Aspect**	**Advantages of paraprobiotics in ice cream**	**Disadvantages of probiotics in ice cream**
Health safety	Paraprobiotics are nonviable, thus eliminating the risks of overgrowth or contamination, making them safer for consumers with health sensitivities [100]	Viable probiotics may pose risks of gastrointestinal discomfort, especially for individuals with compromised immune systems [115]
Stability under freezing and frozen storage conditions of ice cream	Paraprobiotics remain stable under freezing and frozen storage conditions of ice cream, requiring no viability to ensure consistent health benefits throughout storage [14]	Retention of probiotic viability under freezing and frozen storage conditions of ice cream is challenging, with viable counts often decreasing over time, reducing efficacy [110]
Sensory properties	Paraprobiotics do not undergo fermentation, avoiding the production of organic acids (e.g., lactic and acetic acids) or volatile compounds (e.g., acetaldehyde and diacetyl), thus preserving the traditional ice cream flavor, texture, sweetness, and uniformity [100, 104, 114, 116]	Probiotic fermentation produces organic acids (e.g., lactic and acetic acids), volatile compounds (e.g., acetaldehyde and diacetyl), and bitter peptides, leading to sour or vinegary flavors and off-odor that reduce consumer acceptance [97], [109–111], [114]
Immune and gut health	Paraprobiotics confer immune system modulation and gut health benefits, meeting the requirements for functional foods without compromising on safety or quality under freezing conditions [117]	Although live probiotics can support gut health, their viability and stability issues under freezing reduce potential health benefits [110]
Market growth potential	Aligned with the growing demand for health-oriented functional foods, tapping into a projected 37% market growth in ice cream [104]	Market potential for probiotic ice cream is limited by challenges in stability, safety concerns, and fluctuating consumer acceptance [116]

*Note:* In this context, stability refers to the ability of paraprobiotics to maintain their functional properties (e.g., immunomodulatory effects produced by the cell wall components like PGN or EPS) under processing and storage conditions, such as freezing, without requiring live cells. In contrast, viability refers to the survival and metabolic activity of live probiotic cells, which is necessary for their health benefits but difficult to maintain in frozen conditions, due to environmental stresses like low temperatures, pH variations, and oxygen exposure [14, 110]. Live probiotics are viable microorganisms needing survival to confer health benefits, unlike paraprobiotics which are inactivated cells. The 37% projected ice cream market growth refers to Brazil [104].

## Data Availability

The data that support the findings of this study are available from the corresponding author upon reasonable request.
